# Deep structural brain lesions associated with consciousness impairment early after hemorrhagic stroke

**DOI:** 10.1038/s41598-019-41042-2

**Published:** 2019-03-12

**Authors:** Benjamin Rohaut, Kevin W. Doyle, Alexandra S. Reynolds, Kay Igwe, Caroline Couch, Adu Matory, Batool Rizvi, David Roh, Angela Velazquez, Murad Megjhani, Soojin Park, Sachin Agarwal, Christine M. Mauro, Gen Li, Andrey Eliseyev, Vincent Perlbarg, Sander Connolly, Adam M. Brickman, Jan Claassen

**Affiliations:** 1 Department of Neurology, Critical Care Neurology, Columbia University, New York, NY USA; 2 Department of Neurology, The Taub Institute, Gertrude H. Sergievsky Center, Columbia University, New York, NY USA; 3 Mailman School of Public Health, Department of Biostatistics, Columbia University, New York, NY USA; 4 Bioinformatics and Biostatistics Core Facility, iCONICS, IHU-A-ICM, Institut du Cerveau et de la Moelle épinière, Paris, France; 5 Department of Neurosurgery, Columbia University, New York, NY USA

## Abstract

The purpose of this study was to determine the significance of deep structural lesions for impairment of consciousness following hemorrhagic stroke and recovery at ICU discharge. Our study focused on deep lesions that previously were implicated in studies of disorders of consciousness. We analyzed MRI measures obtained within the first week of the bleed and command following throughout the ICU stay. A machine learning approach was applied to identify MRI findings that best predicted the level consciousness. From 158 intracerebral hemorrhage patients that underwent MRI, one third was unconscious at the time of MRI and half of these patients recovered consciousness by ICU discharge. Deep structural lesions predicted both, impairment and recovery of consciousness, together with established measures of mass effect. Lesions in the midbrain peduncle and pontine tegmentum alongside the caudate nucleus were implicated as critical structures. Unconscious patients predicted to recover consciousness by ICU discharge had better long-term functional outcomes than those predicted to remain unconscious.

## Introduction

Insights into mechanisms underlying early and delayed recovery of consciousness following brain injury are limited. Investigators have implicated several subcortical structures to be crucial for maintenance of arousal such as pontine tegmentum, midbrain, basal forebrain, hypothalamus and central thalamus^1–3^. Structures important for conscious processing or awareness include sub-cortical regions (i.e., thalamus, putamen, caudate and pallidum) as well as associative cortical regions (i.e., prefrontal, temporal and parietal cortices) and their connecting neuronal pathways^4–9^. Based on neuropathological, imaging and electrophysiological studies, circuit models have been developed that help conceptualize impairment and recovery of consciousness (e.g., modern concepts of the ascending reticular activating pathway [ARAS]^3,10,11^, mesocircuit model^6,12^ and global neuronal workspace)^5^. Structural damage to regions within this circuitry (e.g., large intracerebral hemorrhage [ICH] in the thalamus) as well as lesions that functionally affect circuit connectivity (e.g., stretching thalamo-cortical projections from mass effect), especially when they are bilateral, may result in clinically indistinguishable unconscious patients. However, depending on the anatomical location and the pathophysiological mechanism of lesions, prognosis for recovery of consciousness can dramatically differ^13^.

Here we studied patients with ICH, a condition that may cause both focal injury to specific subcortical brain regions that are integral parts of the ARAS and/or the mesocircuit model as well as more diffuse injury that can impair network connectivity (e.g., from midline shift and/or edema)^14^. Specifically, we explored how the level of impairment and recovery of consciousness relate to the locations and the extent of subcortical injury quantified by early MRI. We tested the hypothesis that focal lesions within subcortical regions included in the previously mentioned models of consciousness, in addition to established characteristics of the hemorrhage (i.e., volume of ICH and edema, and midline shift [MLS]), contribute to consciousness level during the acute phase of ICH (at the time of MRI and ICU discharge).

## Methods

### Subjects

We studied a prospectively enrolled series of patients with ICH that underwent MRI including fluid attenuated inversion recovery (FLAIR) and diffusion weighted imaging (DWI) within one week of the ICH between March 2009 and November 2015. MRIs were obtained as part of our clinical management protocol whenever feasible. Inclusion criteria were: (1) spontaneous ICH, and (2) MRI obtained within 7 days of the hemorrhage. Exclusion criteria were: (1) age < 18 years, (2) pregnancy, (3) ICH due to tumor, trauma, or hemorrhagic conversion of an ischemic stroke, and (4) patients or families who declined to participate in the study. Patient management was in accordance with current guidelines (Supplementary Material). Data were collected as part of a prospective observational cohort study approved by the local institutional review board (Columbia University Medical Center IRB). Written informed consent was obtained from patients and/or legal surrogates on admission to the ICU, patients that recovered consciousness were given the opportunity to withdraw from the study. All experiments were performed in accordance with relevant guidelines and regulations.

### Clinical variables

We collected baseline demographic and medical history (e.g., age, gender, race), and admission characteristics of the ICH (e.g., ICH volume and location, presumed etiology, intraventricular hemorrhage, primary ICH-score)^15^. We calculated the admission Functional Outcome in Patients With Primary Intracerebral Hemorrhage (FUNC) score by quantifying ICH volume and location, age, Glasgow Coma Scale, and pre-ICH cognitive impairment^16^. Daily assessments included documentation of seizures (as per hospital protocol all unconscious patients undergo continuous EEG monitoring for at least 24 hours)^17,18^, metabolic abnormalities (e.g., renal function and liver failure), and fever. Doses of all sedatives and laboratory values were recorded at the time of all behavioral assessments.

### Behavioral assessment

We assessed level of consciousness daily from ICU admission to ICU discharge, whichever was sooner. As described previously^19^, behavioral assessments of consciousness were performed during morning rounds. These consisted of protocolized, hierarchical assessments categorizing consciousness into three levels of behavioral states: (1) “comatose” (no response to stimulation), (2) “arousable” (opening eyes and/or attending to stimulation), or (3) “conscious” (following simple commands; e.g., “show me two fingers”). To overcome language impairment or aphasia while testing for consciousness, we used in addition to verbal commands, non-verbal cues to induce mimicking (e.g., holding up two fingers and then gesturing to subject’s supported hand). For the classification approach described below, we dichotomized patients into “conscious” (category 3, following verbal and/or non-verbal commands) and “unconscious” (categories 1 and 2; see details in Supplementary Material). According to our ICU protocol daily assessments were performed during interruption of sedation.

### MR acquisition

As part of our clinical protocol we acquired MR images within 7 days of hemorrhage whenever deemed safe by the attending neurointensivist using a 3 T scanner (GE Signa HDx MRI scanner; HD23 software). Total acquisition time did not exceed 45 minutes. We obtained FLAIR, T1-weighted, and DWI sequences (for details please refer to the Supplementary Material).

### Categorization of lesions

Anatomical regions of interest (ROIs) were predefined based on established neuroanatomical atlases^20^ with a focus on subcortical brain regions (henceforth referred to as “subcortical ROIs”) previously implicated in consciousness^1–7,12^. A board-certified neurologist (AR) categorized the presence of blood and perihematomal FLAIR hyperintensity (henceforth referred to as “edema”) for each ROI based on a 3D visualization of FLAIR, T1 and DWI sequences. The following ROIs were included in the models: pontine tegmentum, midbrain (central and peduncles), hypothalamus, basal forebrain, thalamus, pallidum, putamen, and caudate nuclei (see Fig. S1). For purposes of analysis, lesion laterality was reclassified from right/left into ipsi/contralateral using the following approach. The side of the brain with the larger amount of blood was labelled as ipsilateral. The side with the smaller amount was labelled as contralateral. Intraventricular hemorrhage (IVH) was assessed in the 3^rd^, 4^th^, and each lateral ventricle and classified as present or absent. Any challenging cases with bilateral hemorrhage were classified by consensus between three board certified neurologists (AR, JC, BR). A board-certified neurologist (DR) coded the same imaging parameters on a random 20% sample of MRIs blinded to the first coder’s results. Interrater agreement was assessed using kappa statistics.

### Volumetric measurements and midline shift

**Figure 1:**
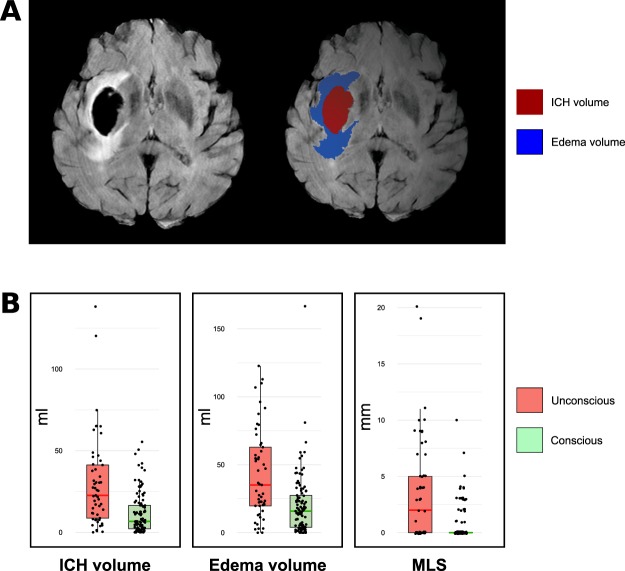
Hemorrhage and edema volumes and midline shift. Panel A. Illustrates the volume on MRIs of one exemplary case. Panel B. Measurements according to consciousness level at time of MRI (normalized values; for details please refer to methods). ICH: Intracerebral Hemorrhage; MLS: midline shift.

Hemorrhage, perilesional edema, and brain volumes were quantified based on FLAIR sequences using a semi-automatized method. Briefly, a gross region-of-interest was identified that encapsulated the affected region (ICH or edema) to automatically compute a 3D image that were visually inspected and manually corrected if necessary (KI, see Supplementary Material and Fig. 1A). Midline shift (MLS) was measured both at the level of the septum pellucidum as well as at the pineal gland, and the larger number was recorded^21^.

### Main outcome

Main outcomes were the level of consciousness observed at time of ICU discharge and the Glasgow Outcome Scale-Extended (GOS-E) obtained 3 months following the hemorrhage via phone interviews^22^. As an additional outcome measure, we recorded the best level of consciousness observed at any time during hospitalization following MRI acquisition.

### Confounders

All patients were clinically evaluated for the presence of seizures, hypo- or hyperglycemia (70 and 200 mg/dL, respectively), hypo- and hypernatremia (133 and 150 mmol/L, respectively), and renal and fulminant liver failure at the time of behavioral assessments. All analyses were directly controlled for potential metabolic confounders (including blood urea nitrogen, creatinine, serum glucose level). In addition to the above outlined protocol of stopping sedation for all behavioral assessments we collected the cumulative doses of all sedative medications administered within the two elimination half-lives preceding clinical assessments^19^.

### Statistical analysis

A machine learning approach using logistic regressions with elastic net regularization was applied to identify the parameters that best predicted consciousness at time of MRI and at time of ICU discharge^23^. This method allows a robust data-driven analysis when there are a large number of features compared to the number of observed events and/or when features are highly correlated. Models were trained on the clinical labels (conscious vs unconscious) obtained either at the time of MRI or at the time of ICU discharge. In order asses robustness of the model, we performed 5-fold cross validations repeated 500 times^24^. Model performance was evaluated using the area under the receiver operating characteristic curve (AUC) with 95% confidence intervals (95% CI). Logistic regression using elastic net regularization were computed with the Glmnet R package (for details please refer to the Supplementary Material).

Differences in baseline features between patients that fulfilled inclusion criteria and those that did not were explored using Fisher’s exact test for categorical and Wilcoxon–Mann–Whitney test for quantitative variables as appropriate. All statistical tests were two-sided. Categorical variables are reported as percentage (number) and quantitative variables as median (interquartile range). Significance was set at P < 0.05. All analyses were performed using the R statistical software version 3.4.1^25^.

## Results

### Enrolment bias analysis

From a total of 690 patients admitted during the study period, 23% (N = 158) had an MRI within the 7 days of the hemorrhage and fulfilled the inclusion criteria. Patients included in the study more frequently had presumed amyloid as the underlying etiology, lobar location, better admission GCS, primary ICH and FUNC scores, smaller ICH volumes on the admission CT scan, and better outcomes as reflected in the 3-months GOS-E, when compared to patients that were not included (Table 1). MRI scans were obtained within a median of 2 (IQR 1, 3) days from ICH. Main ICH etiologies were hypertension (49%; N = 78) and amyloid (37%; N = 59).

**Table 1:** Baseline characteristics of the reported cohort and enrolment bias.

	MRI (N = 158)	No MRI (N = 532)	p-value
Age, years	68 [54, 77]	63 [50, 76]	0.05
Female	71 (45)	244 (47)	0.6
White	51 (32)	148 (29)	0.4
Presumed etiology			<0.01
Hypertensive	78 (49)	241 (48)	
Amyloid	59 (37)	62 (12)	
Coagulopathy*	16 (10)	86 (17)	
Other	5 (3)	121 (24)	
GCS at admission	14 [9, 15]	10 [5, 15]	<0.01
**ICH characteristics on admission CT**			
Lobar	56 (35)	110 (21)	<0.01
Deep	62 (39)	240 (47)	0.1
Infratentorial	28 (18)	74 (15)	0.2
ICH volume			<0.01
<30	121 (78)	306 (66)	
30–60	27 (17)	89 (19)	
>60	7 (5)	69 (15)	
IVH	72 (47)	281 (59)**	<0.01
**ICH prognostic scores**			
ICH score	1 [1, 2]	2 [1, 3]	<0.01
FUNC score	9 [7, 10]	8 [6, 9]	<0.01
**Hospital course**			
EVD	31 (20)	142 (27)	0.05
Clot evacuation	16 (10)	69 (13)	0.3
ICU stay, days	4 [2, 8]	4 [2, 9]	0.06
Hospital stay, days	10 [6, 20]	8 [5, 19]	0.05
**Outcome 3 months**			
GOS	3 [2, 4]	3 [2, 5]	<0.01
Dead	24 (22)	158 (42)**	<0.01

Data reported as n (%) or median [25%-IQR, 75%-IQR] as appropriate.

MRI: Magnetic Resonance Imaging; GCS: Glasgow Coma Scale; ICH: Intracerebral Hemorrhage; CT: Computed Tomography; IVH: Intraventricular Hemorrhage; EVD: External Ventricular Drain; ICU: Intensive Care Unit; GOS: Glasgow Outcome Scale; GOS-E: Glasgow Outcome Scale – Extended. *Coagulopathy, primary hematological disorder and medication induced combined; **more than 5% missing data.

### Patient cohort

**Figure 2:**
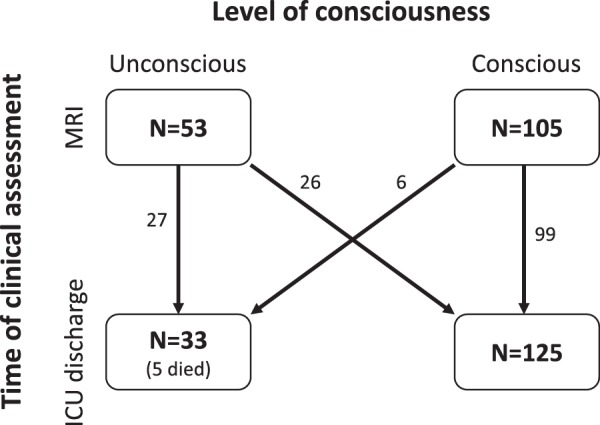
Flow chart. Level of consciousness assessed at MRI and ICU discharge. Note that for the 5 patients who died in the ICU, we considered the last neurological exam as the assessment at ICU discharge (of those that died, 3 patients were unconscious and 2 conscious at time of MRI, all of them were unconscious prior to death).

From a total of 158 ICH patients, 66% (N = 105) were conscious, and 34% (N = 53) were unconscious at the time of MRI Fig. 2). At ICU discharge (occurring on median day 4 [2, 8]), 79% (N = 125) were conscious, 18% (N = 28) remained unconscious, and 3% were dead (N = 5). 49% (N = 26) of initially unconscious patients recovered consciousness at ICU discharge. 6% (N = 6) of the initially conscious patients became unconscious during the ICU stay. Reasons for secondary unconsciousness included worsening edema (N = 3), hydrocephalus (N = 1), ventriculitis (N = 1), and seizures (N = 1). At the time of MRI acquisition and ICU discharge, hypo- or hyperglycemia, hypo- or hypernatremia, renal or fulminant liver failure were not present to explain unconsciousness. One patient with secondary loss of consciousness was seizing prior to death (for the purposes of the study this was considered the ICU discharge time).

At time of MRI scan only 15% (N = 24) of patients received any sedative medication (see Table S1) and none of the patients received sedatives at ICU discharge. In patients that were unconscious at the time of MRI, propofol had been administered more frequently (19% vs 6%) but at lower cumulative doses (131 ± 69 vs 361 ± 202 mg over the 2 preceding half-lives) than those that were conscious.

### MRI findings

**Figure 3:**
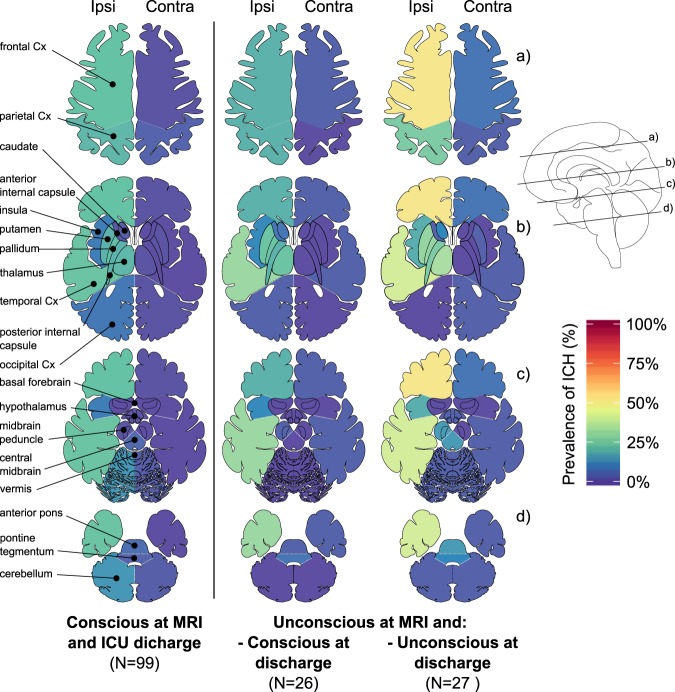
Location of hemorrhages according to consciousness level. Prevalence of ICH observed on MRI are shown by level of consciousness at the time of the MRI and on hospital discharge in three groups: (1) patients that were conscious both at time of MRI and at discharge, (2) patients that were unconscious at the time of MRI and were conscious by the time of ICU discharge, (3) and patients that were unconscious at the time of MRI and remained unconscious by the time of ICU discharge. Patients that were conscious at the time of MRI but unconscious on ICU discharge are not displayed (N = 6). (“unconscious”: patients did not follow or mimic even simple commands; “conscious”: patients followed or mimicked simple commands. “ipsi” and “contra” stand for ipsilateral and contralateral with respect to the primary side of the hemorrhage; Cx: cortex.

73% (N = 116) of patients had an isolated unilateral supratentorial ICH whereas 11% (N = 17) had an isolated infratentorial ICH and 7% (N = 11) had both (supratentorial and infratentorial ICH). ICH was most frequently observed in frontal (37%) and temporal cortices (27%), globus pallidus (23%), thalamus (23%), putamen (22%), posterior limb of internal capsule (21%), and the parietal cortex (22%). Distribution of lesions observed among conscious and unconscious patients are displayed in Fig. 3. Details regarding lesion in all ROIs are available in Tables S2 and S3. Interrater agreements for both ICH and edema measures in all ROIs reached a median kappa of 0.82 (0.66, 0.88).

### Prediction of consciousness at time of MRI and ICU discharge

The algorithms trained on subcortical ROIs together with ICH and edema volumes, MLS and IVH accurately predicted level of consciousness both at the time of MRI and at ICU discharge (AUC = 0.74 [95%CI: 0.72, 0.75] and 0.74 [95%CI: 0.73, 0.75], respectively). ICH volume and MLS were the most important predictors (Table 2). Lesions in pontine tegmentum (edema), in the ipsilateral caudate nuclei (ICH and edema), as well as in the contralateral midbrain peduncle, putamen and pallidum (edema), were identified as predictors of being unconscious at time of MRI (Table 2; Fig. S2). Lesions in pontine tegmentum (edema), ipsilateral midbrain peduncle (ICH) and ipsilateral caudate nuclei (ICH and edema; please refer to the Supplementary Material for details on these patients) were identified as predictors of being unconscious at ICU discharge (Table 2; Fig. S3). In addition, over both models, a few ROIs were found to be associated with being conscious (e.g., pallidum, central midbrain, basal forebrain; see Table 2, Figs S2 and S3 and discussion).

**Table 2:** Weights of models predicting consciousness at time of MRI and at time of ICU discharge.

Subcortical ROIs	Conscious at time of MRI		Conscious at time of ICU discharge	
	ICH	Edema	ICH	Edema
Caudate ipsi	**−0**.**12 [−0**.**7**, **0]**	**−0**.**32 [−0**.**65**, **0]**	**−0**.**78 [−1**.**14**, **−0**.**25]**	**−0**.**72 [−0**.**99**, **−0**.**48]**
Caudate contra	0 [0, 0]	0 [0, 0]	0 [0, 0]	0 [0, 0]
Putamen ipsi	0 [0, 0]	0 [0, 0]	0 [0, 0]	0 [0, 0]
Putamen contra	**0 [−0**.**03**, **0]**	**0 [−0**.**32**, **0]**	0 [0, 0]	0 [0, 0]
Pallidum ipsi	0 [0, 0]	**0**.**31 [0**, **0**.**72]**	0 [0, 0]	0 [0, 0]
Pallidum contra	**0 [0**, **0**.**67]**	**0 [−0**.**82**, **0]**	0 [0, 0]	0 [0, 0]
Thalamus ipsi	**0 [−0**.**06**, **0]**	0 [0, 0]	0 [0, 0]	0 [0, 0]
Thalamus contra	0 [0, 0]	0 [0, 0]	0 [0, 0]	0 [0, 0]
Basal forebrain	0 [0, 0]	0 [0, 0]	0 [0, 0]	**0 [0**, **0**.**42]**
Hypothalamus	0 [0, 0]	**0 [−0**.**06**, **0]**	0 [0, 0]	0 [0, 0]
Midbrain peduncule ipsi	0 [0, 0]	0 [0, 0]	**0 [−0**.**12**, **0]**	0 [0, 0]
Midbrain peduncule contra	0 [0, 0]	**−1**.**05 [−1**.**75**, **0]**	0 [0, 0]	**0 [0**, **0**.**73]**
Central midbrain	**0**.**6 [0**, **1**.**97]**	0 [0, 0]	0 [0, 0]	0 [0, 0]
Pontine tegmentum	0 [0, 0]	**−0**.**05 [−1**.**2**, **4 0]**	0 [0 0]	**−0**.**86 [−1**.**59–0**.**21]**
Diffuse injuries*				
Volume	**−3**.**57 [−4**.**9**, **−1**.**92]**	**−0**.**94 [−1**.**74**, **−0**.**01]**	**−3**.**5 [−4**.**53–2**.**7]**	**−0**.**87 [−1**.**54**, **−0**.**34]**
MLS	**−2**.**94 [−4**.**41**, **−1**.**64]**		**−1**.**12 [−1**.**75**, **0**.**51]**	
IVH	0 [0, 0]		**−0**.**58 [−0**.**79**, **−0**.**41]**	

Data given as median [25%-IQR, 75%-IQR] of the weights obtained over the 500 cross validation iterations. A high value (either positive or negative) indicates that the feature is relevant for classification (Negative values indicate prediction of being unconscious, positive values of being conscious). A value close to zero indicates that the feature was not relevant for classification.

ROI: Region of Interest; ispi: ipsilateral; contra: contralateral; MLS: Midline shift; ICH: Intracerebral Hemorrhage; IVH: Intraventricular Hemorrhage. *Volume weights correspond to 10 ml units, MLS weights correspond to 1 mm changes and IVH was dichotomized as present or absent.

Patients unconscious at time of MRI that were predicted to be conscious at ICU discharge more frequently were arousable than those that were predicted to remain unconscious, although this difference did not reach statistical difference (61% [N = 25/41] vs 33% [N = 4/12]; p-value = 0.1). In univariate analysis, GCS and FUNC scores were associated with level of consciousness at time of MRI and ICU discharge but primary ICH score was only associated with level of consciousness at ICU discharge (Table S4).

### Confounders

The model trained to predict consciousness at the time of MRI retained cumulative doses of midazolam and fentanyl together with the imaging parameters (Supplementary Material). However, performance of the models was not improved by including sedative doses (cumulative or current), cortical and cerebellar ROIs, or measures of metabolic disarray (i.e., renal insufficiency, glucose level).

### Functional outcome at 3 months

**Figure 4:**
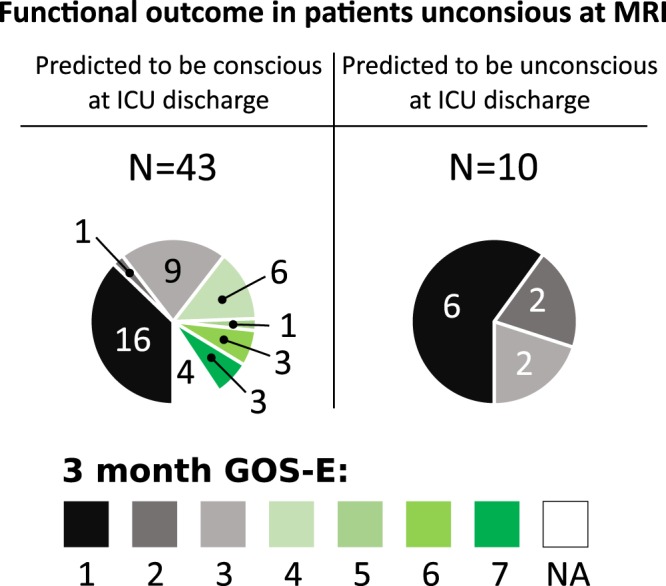
Functional outcome in patients unconscious at MRI. Displayed are the 3-month GOS-E scores in unconscious patients that were predicted to be conscious or unconscious at ICU discharge. Unconscious patients at time of MRI that were predicted to be conscious at ICU discharge (N = 43) based on imaging findings were more likely to be conscious at ICU discharge and had a greater chance to reach a GOS-E ≥ 4 at 3 months (illustrated in shades of green; p-value = 0.02). GOS-E: Glasgow Outcome Scale – revised; NA: not available (lost follow-up).

Three-month GOS-E were obtained for 92% (N = 145/158) of the patients. Mean GOS-E was 4 (IQR 1, 6; Table 1), with 53% (N = 77/145) of patients having a favorable outcome (GOS-E ≥ 4: 4% GOS-E 8, 19% GOS-E 7, 14% GOS-E 6, 1% GOS-E 5, 14% GOS-E 4), 19% (N = 27/145) in a vegetative or totally dependent state (3% GOS-E 2, 15% GOS-E 3), and 28% of patients being dead (N = 41/145; GOS-E 1). FUNC score reliably predicted functional independence (GOS-E ≥ 4) at month 3 (32% (N = 6/19) with a FUNC score ≥ 9 vs 3% (N = 1/30) for those with a FUNC score of < 9; p-value = 0.01). Patients that were unconscious at the time of MRI but predicted to be conscious at ICU discharge based on our model, more frequently were observed conscious at any time in the 30 days following the MRI (54% [N = 22/41] vs 33% [N = 4/12]; p-value = 0.3) and had better 3-month functional outcomes than those that were predicted to still be unconscious at ICU discharge (GOS-E ≥ 4: 35% (N = 13/37) vs 0% (N = 0/12) vs; p-value = 0.02; Fig. 4).

## Discussion

More than half of ICH patients that are unconscious on admission are dead at one year after the bleed, and even though our ability to accurately prognosticate recovery is dismal, the primary mode of death is withdrawal of life sustaining therapies^26^. Accurate prediction of functional outcomes is challenging in the acute setting and confounded by biases contributing to the self-fulfilling prophecy of poor outcomes^27,28^. In this study we show that lesions identified on MRIs obtained within one week of ICH not only correlate with level of consciousness at the time of MRI but, more importantly, are able to identify patients that will recover consciousness prior to ICU discharge and have better 3-month functional outcomes. The identified predictors confirm previous findings (ICH volume, MLS and IVH) but also provide new insight on subcortical structures implicated in the physiology of consciousness^1–7,12^.

We confirm that measures reflecting the impact on both hemispheres (i.e., ICH and edema volumes, MLS and IVH) are major determinants of impairment and recovery of consciousness, survival and functional outcomes. ICH volume is a well-known prognostic factor for both 30-day mortality and 90-day disability^16,28^. MLS is linked to high ICH and edema volume and is clinically associated with herniation. Both large ICH volumes and MLS are seen in patients with increased intracranial pressure. All of these variables may cause bilateral impairment of widespread brain regions, which frequently is associated with unconsciousness^2^.

Additionally, we identified three main subcortical structures implicated with consciousness impairment. These findings further support the role of the pontine tegmentum and midbrain peduncles, which have been implicated reliably in several clinical studies^1–3,7^. Interestingly, the caudate nucleus, which has been implicated in wakefulness in the rat^29^, is also included in many models of human consciousness as part of the frontal cortical–striatopallidal–thalamocortial loop systems^2,4,6^. According to the mesocirctuit theory, a decrease in the indirect excitatory activity of the Medium Spiny Neurons of the caudate and the putamen nuclei (special type of GABAergic inhibitory neurons) on the thalamus could explain an alteration of consciousness^6^. Hypometabolism of the caudate has been reported in unconscious patients^30^ and, the caudate nucleus atrophies in chronic disorders of consciousness^8,31,32^. Reports of isolated bilateral caudate lesions are very rare but have been seen in patients with impairment of consciousness ranging from disorientation and confabulations to somnolence^33,34^. Caudate ICH has been previously associated with impairment of consciousness and cognition^35^, however, since the caudate nucleus forms the wall of the lateral ventricle, it frequently is associated with IVH, hampered causal inference^36^. In our study, the majority of the patients with caudate lesions also had IVH. However, these patients were less likely to be conscious, both at time of MRI and ICU discharge, than patients with IVH in the absence of a caudate lesion (see Supplementary Material). At a minimum, caudate lesions appear to play a mediating effect on the relationship between IVH and impairment of consciousness. Supporting a recent paper^37^ we did not find evidence of thalamic lesions associated with impairment of consciousness. Finally, it is worth noting that the weighs attributed to caudate lesions were systematically greater than for IVH. In light of these findings, the present study provides further support implicating lesions of the caudate nucleus in impairment and early recovery of consciousness, independently from the frequently associated IVH.

The patient cohort studied here is not necessarily representative for all ICH patients as our enrolment bias analysis illustrates. Patients that were included tended to have slightly less neurological impairment, smaller hemorrhages, amyloid etiology, and better outcomes. This is likely a reflection of provider safety concerns for MRI scanning and family preferences.

This study has several limitations. First, assessment of consciousness relied on a previously described, standardized neurological assessment^19^ instead of a scale specifically developed for the assessment of consciousness such as the Coma Recovery Scale Revised (CRS-R)^38^. However, the CRS-R has some limitations in the ICU setting as it was primarily developed for patients in the subacute and chronic rehabilitation setting. Assessments with the CRS-R are time consuming posing a challenge in a hectic ICU environment during which patients consciousness level often fluctuates. We acknowledge that this assessment of consciousness will likely underestimate the presence of conscious and does not capture patients with cognitive motor dissociation^39,40^. Second, assessments of consciousness in patients with aphasia and delirium may be challenging. To capture nonverbal command following in aphasic patients we assessed, both verbal and non-verbal (i.e. mimicking) commands. Delirium in general and hypoactive delirium in particular are common in acutely brain injured patients and can interact with consciousness assessments. This confounder will affect any behavioral assessment in brain injured patients including the CRS-R^41^. However, the vast majority of patients with hyperactive delirium would be expected to demonstrate at least intermittent command following. Third, sedation is frequently used in the critical care setting and can confound assessments of consciousness. We minimized doses of sedation as recommended in guidelines^42^ and systematically accounted for sedation given at the time of and preceding the assessment at time of MRI. Note that this limitation only applies the model trained at time of MRI since none of the patients received sedation at time of ICU discharge. Fourth, MRI based assessments of hemorrhage can be challenging as MRI signal changes are observed over time^43^. Subacute hemorrhages typically appear as hypointense signal in FLAIR sequences between 2–7 days, which was within the inclusion criterion in this study. Finally, confirmatory investigations to validate our findings on an independent dataset will be necessary in future studies.

Taken together, our results suggest that measures of injury obtained from routine clinical MRI sequences may allow to predict failure to recover consciousness by ICU discharge and functional outcomes in patients with acute brain injury more accurately. Focal lesions in key structures within previously described models of consciousness together with measures related to mass effect of the hemorrhage predict early recovery of consciousness. Both, adding a comprehensive assessment of structural connectivity between these key structures (i.e., using diffusion tensor imaging analysis)^44^ as well as quantifying functional connectivity (using functional imaging or EEG markers) may further strengthen the accuracy of this model.

## Supplementary information

Supplementary Material
