# Supplementary material for: Deep structural brain lesions associated with consciousness impairment early after hemorrhagic stroke
Source: Sci Rep. 2019 Mar 12;9:4174. doi: 10.1038/s41598-019-41042-2 (PMC6414498; doi:10.1038/s41598-019-41042-2)
Supplement: Supplementary file 1 — Supplementary Material [file 41598_2019_41042_MOESM1_ESM.pdf]

# Supplementary Material

**Deep structural brain lesions associated with consciousness impairment early after hemorrhagic stroke.**

Benjamin Rohaut, Kevin W. Doyle, Alexandra S. Reynolds, Kay Igwe, Caroline Couch, Adu Matory, Batool Rizvi, David Roh, Angela Velasquez, Murad Megjhani, Soojin Park, Sachin Agarwal, Christine M. Mauro, Gen Li, Andrey Eliseyev, Vincent Perlbarg, Sander Connolly, Adam M. Brickman, Jan Claassen.

## Methods

### General Management

Patients' management was in accordance with the American Heart Association guidelines including strict blood pressure control, reversal of anticoagulation, and treatment of hydrocephalus[1]. Sedation was maintained at the minimum level to ensure comfort and safety and as recommended by guidelines; daily interruption was performed when deemed safe[2,3].

### Behavioral assessment

To facilitate the contextualization of the behavioral assessment used in the present study within the framework of the large body of evidence on chronic disorders of consciousness, we provide here a brief comparison with the established taxonomy. According to the CRS-R, the applied cut-off (patients that respond to commands inconsistently or consistently would correspond to the limit between the Minimally Consciousness State "plus" (MCS "plus") and MCS "minus", even though this definition fluctuates[4]. Comatose, unresponsive wakefulness syndrome[5] and MCS

“minus” patients (patients that at best demonstrate visual pursuit or fixation) were categorized as unconscious, even though some of these patients may have had more complex cognition than suggested by their behavior (this state is also known as “cognitive motor dissociation”)[6,7].

## MR acquisition

All MRIs were obtained on a 3T scanner (GE Signa HDx MRI scanner; HD23 software), which is subject to monthly quality controls using an American College of Radiology phantom. Patient vital signs were carefully monitored throughout the acquisition and supervised by a radiology technician, a critical care nurse or physician, and a respiratory therapist. MRI scans were immediately aborted in cases of patient instability (e.g., desaturation). We obtained FLAIR, T1-weighted, and DWI sequences. Sequence parameters were set as follow: for FLAIR: repetition time = 8000 ms, echo time = 70 ms, flip angle = 130 degrees, field of view = 256 x 256 matrix with a 1 mm slice thickness; for T1-weighted: repetition time =3042.78 ms, echo time = 17.6 ms, flip angle = 90 degrees, field of view = matrix 288 x 224 with a 4 mm slice thickness; and for DWI: repetition time = 7000 ms, echo time = 73.1 ms, flip angle = 90 degrees, field of view = 128 x 192 with a 5 mm slice thickness.

## Volumetric measurements and midline shift

Each patient’s FLAIR images were skull stripped and manually edited to remove non-brain tissue[8]. Hemorrhage, perilesional edema, and brain volumes were quantified based on these FLAIR sequences. Using 3D Slicer, a gross region-of-interest was identified that encapsulated the affected region[9]. Next, lower and upper intensity values were set to label voxels within areas of edema, which appeared as hyperintense, and to label voxels within the hemorrhagic lesion, which appeared as

hypointense. Labelled images were visually inspected and manually corrected if necessary (KI). Hemorrhage and edema volumes were calculated by summing the respectively labelled voxels and multiplying the values by the voxel dimensions (see Figure 1 panel A). Midline shift (MLS) was measured both at the level of the septum pellucidum as well as at the pineal gland, and the larger number was recorded[10–12]. Normalization of volumes and MLS: Individual hemorrhage and edema volumes as well as midline shift were normalized using the patient's individual total brain volume in relation to the overall group mean brain volumes, measured across all subjects.

Individual hemorrhage and edema volumes as well as midline shift were normalized using the patient's individual total brain volume in relation to the overall group mean brain volumes, measured across all subjects.

## Statistical analysis

Machine learning approach. We applied logistic regression allowing data-driven features selection using the elastic net regularization method[13]. This analysis intended to explore whether impairment and recovery of commands following primarily relates to injury in subcortical ROIs or to global hemorrhage characteristics (including hemorrhage volume, surrounding edema volume, and midline shift). The elastic net regularization method comprises a Least Absolute Shrinkage And Selection Operator (LASSO) penalty ($L_1$) and Ridge penalty ($L_2$). The LASSO penalty is a strict penalty that performs feature selection by shrinking coefficients not associated with commands following to zero, while the Ridge penalty shrinks the coefficients of correlated features towards their average[13]. In this model, a parameter λ controls the model complexity with higher values resulting in a less complex model (fewer number of variables) and α controls the balance between two types of model

complexity penalties, including the Ridge (α =0) and the Lasso penalties (α=1). Values for λ were internally optimized using a 10-fold cross validation. We set α=0.95, corresponding to an elastic net which corresponds to the best approach when there are a large number of features compared to the number of observed events and/or when features are highly correlated.

Models were trained on the clinical labels (conscious vs unconscious) obtained either at the time of MRI or at the time of ICU discharge. In order to obtain reliable estimates of model accuracy, the previously described training procedures were performed 500 times using for each iteration an 80% randomly selected subset of the data and testing the resulting model on the 20% holdout set[14]. Proportions of commands following patients were kept constant in both trainings and test sets and were equal to the proportion in the whole dataset. Each model performance was evaluated using the area under the receiver operating characteristic curve (AUC). For each iteration of a model, parameters' weights and AUCs were computed. All the AUCs obtained during the cross-validation process were summarized by the mean reported with 95% confidence intervals (95% CI). Parameter weights were aggregated and reported as median (interquartile range). Logistic regression using elastic net regularization were computed with the Glmnet R package[13] https://rdrr.io/cran/glmnet/man/glmnet.html). In all models, continuous variables were normalized using z-scores.

# Results

## Characteristic of patients with caudate lesions

Twenty-three patients presented with a lesion in the caudate nucleus: 10 had ICH associated with edema and 13 edema without ICH. The comparison of

consciousness level at time of MRI and ICU discharge between this subgroup and the rest of the cohort is provided in the Table S5. While 91% (N=21) of patients with a caudate lesion also had IVH (compared to 41% N=56 in patients without caudate lesions; Fischer's test p-value <0.01) they were less likely to be conscience both at time of MRI and ICU discharge than patients with IVH without caudate lesion (Fischer's test p-value = 0.2 and 0.1, respectively). This observation further supports the importance of caudate lesions for impairment of consciousness amongst patients with IVH.

## Characteristic of patients with brainstem lesions

A recent report on 12 comatose patients has suggested that left pontine tegmentum lesions could carry a worse prognosis[15]. Since we merged data as ipsi/contralateral, our methodology was not designed to the exploration of the impact of laterality. However, over the 4 patients that had isolated brainstem hemorrhage that involved the pontine tegmentum, both patients that had an isolated right ICH recovered consciousness whereas only 1 out of 2 with isolated left ICH recovered consciousness.

## Confounders

In addition to the 2 models reported in the main manuscript we also ran two additional models that included all confounders. In addition to subcortical ROIs with ICH and edema volumes, MLS and IVH (models reported in the main manuscript) these models also accounted for cortical and cerebellar ROIs, metabolic derangement (i.e., renal insufficiency, glucose level) and sedative doses (sedative confounders only apply for the model predicting consciousness at time of MRI as none of the patients were sedated at time of ICU discharge). Performance tended to

deteriorate with inclusion of these additional parameters (AUC = 0.73 [95%CI: 0.73, 0.74] and 0.70 [95%CI: 0.69, 0.71], respectively). Weighs are reported in Figure S4 and S5. Even in the additional models the vast majority of variables selected in the main models remained predictive. At time of MRI, volumes, IVH, MLS pontine tegmentum (edema), ipsilateral caudate nucleus (ICH and edema), and contralateral midbrain peduncle (edema) remained in the model, suggesting a robust effect. The main additional selected parameters were contralateral temporal edema followed by ipsilateral temporal ICH. Similarly, at time of ICU discharge volumes, IVH, MLS alongside with pontine tegmentum (edema) and ipsilateral caudate nuclei (ICH and edema)) were again selected. The main additional selected parameters were lesions in the contralateral temporal cortex wand in the ipsilateral frontal cortex.

# Supplemental references


1. Hemphill, J. C. *et al.* Guidelines for the Management of Spontaneous Intracerebral Hemorrhage: A Guideline for Healthcare Professionals From the American Heart Association/American Stroke Association. *Stroke* **46**, 2032–2060 (2015).
2. Barr, J. *et al.* Clinical Practice Guidelines for the Management of Pain, Agitation, and Delirium in Adult Patients in the Intensive Care Unit. *Critical Care Medicine* **41**, 278–280 (2013).
3. Oddo, M. *et al.* Optimizing sedation in patients with acute brain injury. *Crit Care* **20**, 128 (2016).
4. Bruno, M.-A., Vanhaudenhuyse, A., Thibaut, A., Moonen, G. & Laureys, S. From unresponsive wakefulness to minimally conscious PLUS and functional locked-in syndromes: recent advances in our understanding of disorders of consciousness. *J Neurol* **258**, 1373–1384 (2011).
5. Laureys, S. *et al.* Unresponsive wakefulness syndrome: a new name for the vegetative state or apallic syndrome. *BMC Med* **8**, 68 (2010).
6. Schiff, N. D. Uncovering hidden integrative cerebral function in the intensive care unit. *Brain* **140**, 2259–2262 (2017).
7. Rohaut, B., Eliseyev, A. & Claassen, J. Uncovering consciousness in unresponsive ICU patients: technical, medical and ethical consideration. *Critical Care* (2019). doi:(in press)
8. Smith, S. M. Fast robust automated brain extraction. *Hum. Brain Mapp.* **17**, 143–155 (2002).
9. Fedorov, A. *et al.* 3D Slicer as an image computing platform for the Quantitative Imaging Network. *Magnetic Resonance Imaging* **30**, 1323–1341 (2012).
10. Ropper, A. H. Lateral displacement of the brain and level of consciousness in patients with an acute hemispheral mass. *N. Engl. J. Med.* **314**, 953–958 (1986).

11. Zazulia, A. R., Diringer, M. N., Derdeyn, C. P. & Powers, W. J. Progression of mass effect after intracerebral hemorrhage. *Stroke* **30**, 1167–1173 (1999).

12. Yang, W.-S. *et al.* Defining the Optimal Midline Shift Threshold to Predict Poor Outcome in Patients with Supratentorial Spontaneous Intracerebral Hemorrhage. *Neurocrit Care* (2017). doi:10.1007/s12028-017-0483-7

13. Friedman, J., Hastie, T. & Tibshirani, R. Regularization Paths for Generalized Linear Models via Coordinate Descent. *J Stat Softw* **33**, 1–22 (2010).

14. Kriegeskorte, N., Simmons, W. K., Bellgowan, P. S. F. & Baker, C. I. Circular analysis in systems neuroscience: the dangers of double dipping. *Nat. Neurosci.* **12**, 535–540 (2009).

15. Fischer, D. B. *et al.* A human brain network derived from coma-causing brainstem lesions. *Neurology* **87**, 2427–2434 (2016).

1 ## Supplemental figures

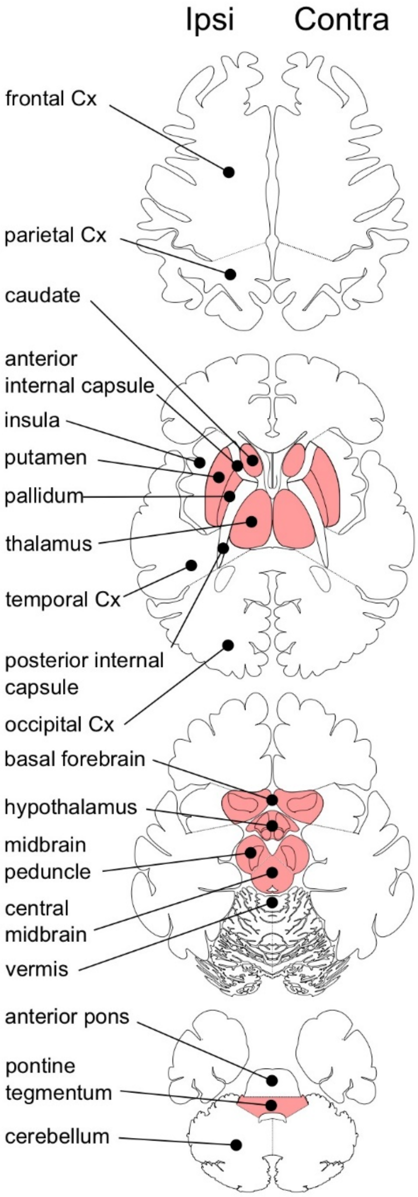


2
3 **Figure S1. Anatomical segmentation and subcortical ROIs I**. Anatomical
4 segmentation is represented with the explored subcortical regions of interest (ROIs)
5 in pink.

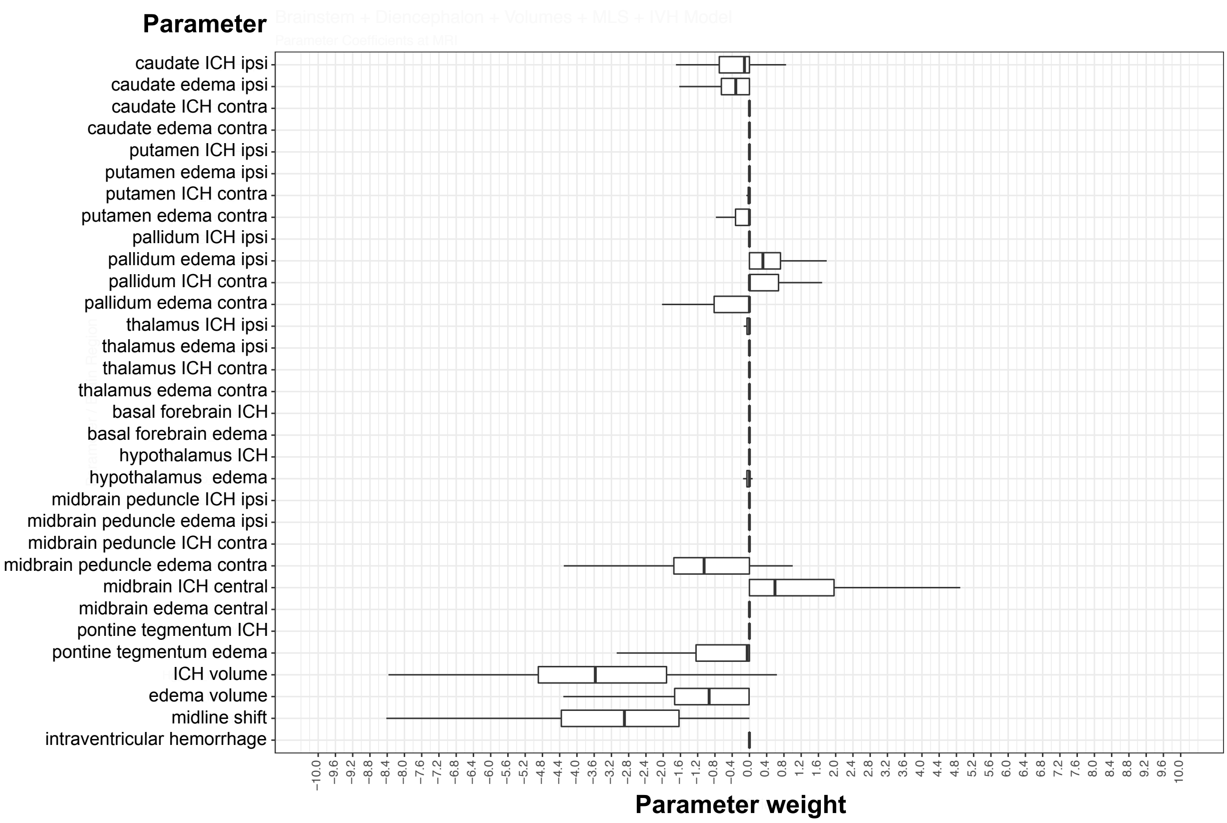


**Figure S2. Weights of the model predicting consciousness at time of MRI.**

Include features were: subcortical ROIs with ICH and edema volumes, MLS and IVH. Boxplot of all parameter weights obtained over the 500 cross-validation iterations. For scaling visualization purpose outliers are not shown in this figure. Negative weight value corresponds to a predictive value for being unconscious, positive weight corresponds to a predictive value for being conscious.

ICH: Intracerebral Hemorrhage; IVH: Intraventricular Hemorrhage; “ipsi" and "contra" stand for ipsilateral and contralateral with respect to the primary side of the hemorrhage.

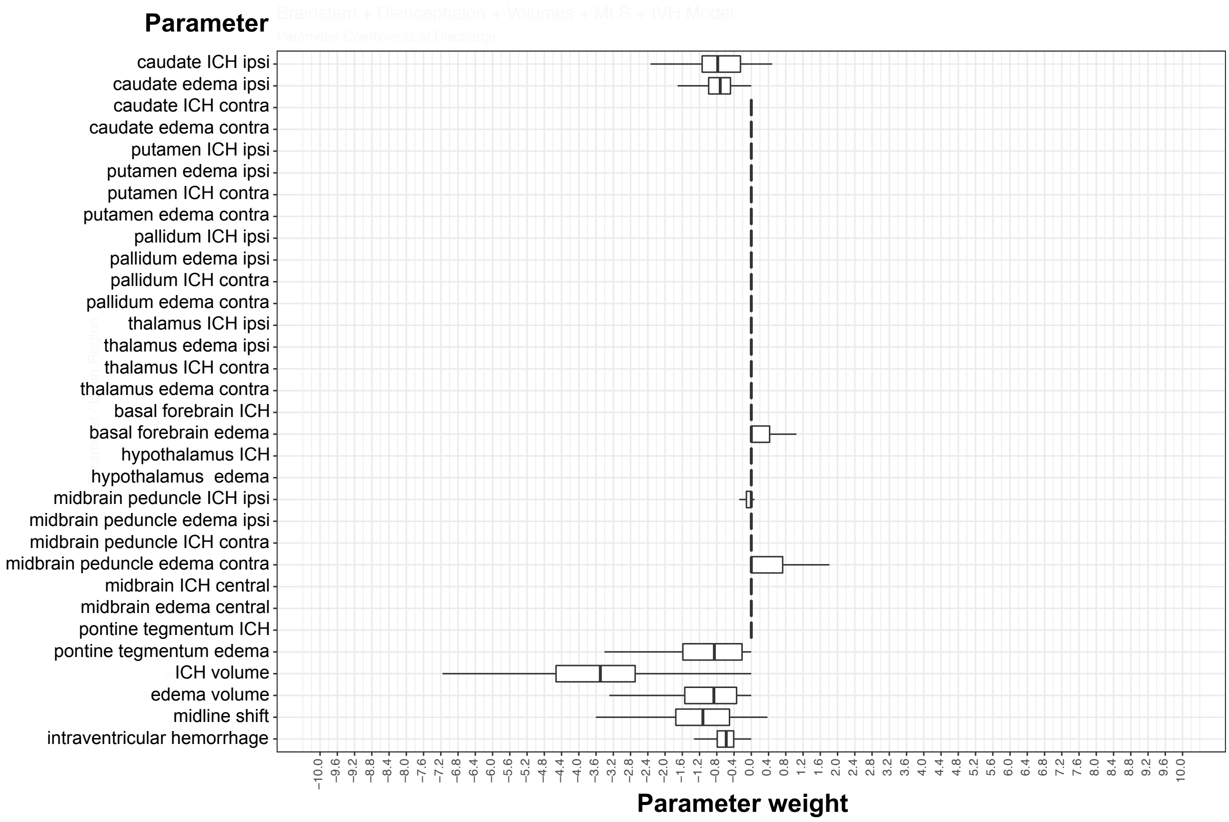


**Figure S3. Weights of the model predicting consciousness at time of ICU discharge.**

Include features were: subcortical ROIs with ICH and edema volumes, MLS and IVH. Boxplot of all parameter weights obtained over the 500 cross-validation iterations. For scaling visualization purpose outliers are not shown in this figure. Negative weight value corresponds to a predictive value for being unconscious, positive weight corresponds to a predictive value for being conscious.

ICH: Intracerebral Hemorrhage; IVH: Intraventricular Hemorrhage; "ipsi" and "contra" stand for ipsilateral and contralateral with respect to the primary side of the hemorrhage

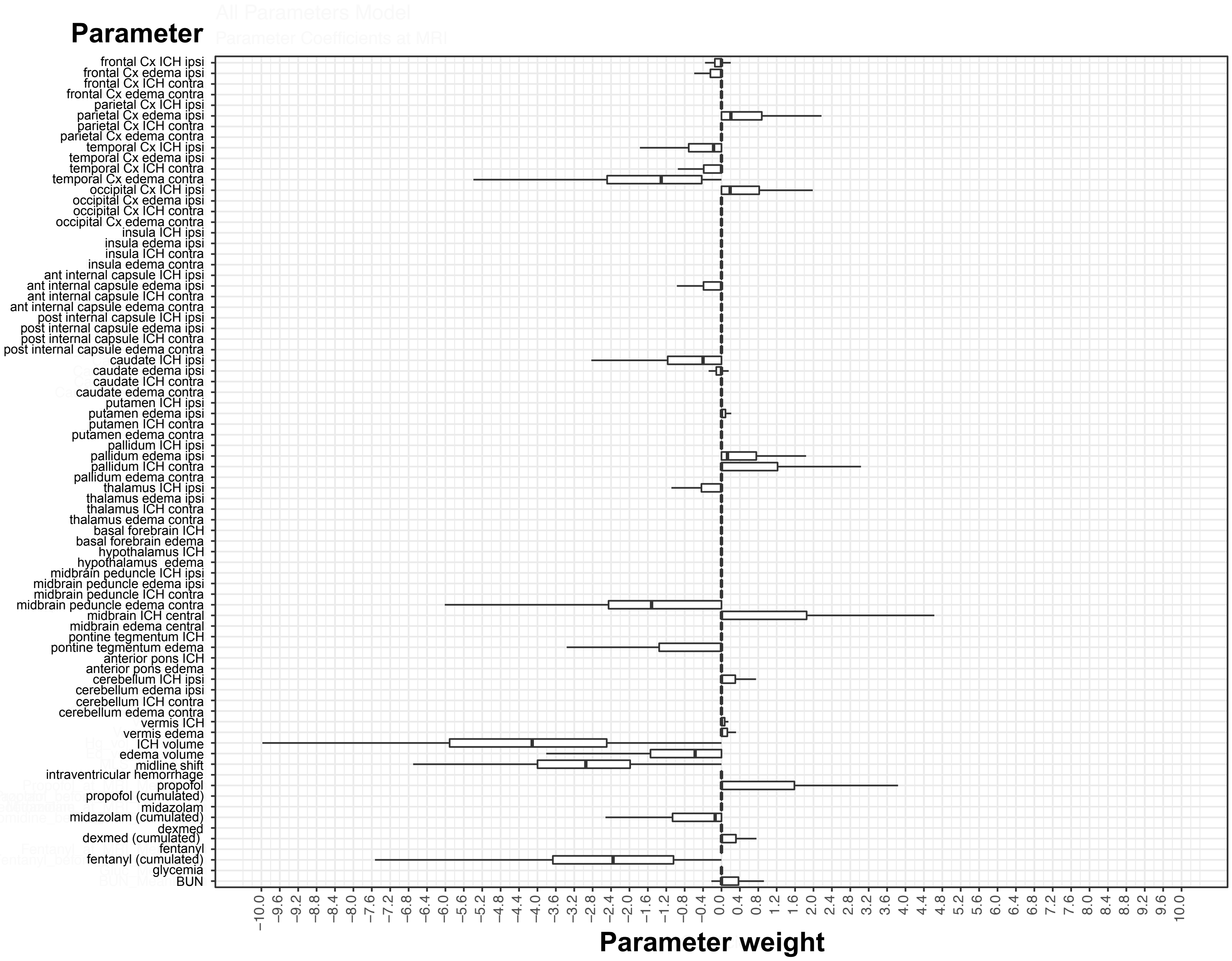
Parameter
frontal Cx ICH ipsi
frontal Cx edema ipsi
frontal Cx ICH contra
frontal Cx edema contra
parietal Cx ICH ipsi
parietal Cx edema ipsi
parietal Cx ICH contra
parietal Cx edema contra
temporal Cx ICH ipsi
temporal Cx edema ipsi
temporal Cx ICH contra
temporal Cx edema contra
occipital Cx ICH ipsi
occipital Cx edema ipsi
occipital Cx ICH contra
occipital Cx edema contra
insula ICH ipsi
insula edema ipsi
insula ICH contra
insula edema contra
ant internal capsule ICH ipsi
ant internal capsule edema ipsi
ant internal capsule ICH contra
ant internal capsule edema contra
post internal capsule ICH ipsi
post internal capsule edema ipsi
post internal capsule ICH contra
post internal capsule edema contra
caudate ICH ipsi
caudate edema ipsi
caudate ICH contra
caudate edema contra
putamen ICH ipsi
putamen edema ipsi
putamen ICH contra
putamen edema contra
pallidum ICH ipsi
pallidum edema ipsi
pallidum ICH contra
pallidum edema contra
thalamus ICH ipsi
thalamus edema ipsi
thalamus ICH contra
thalamus edema contra
basal forebrain ICH
basal forebrain edema
hypothalamus ICH
hypothalamus edema
midbrain peduncle ICH ipsi
midbrain peduncle edema ipsi
midbrain peduncle ICH contra
midbrain peduncle edema contra
midbrain ICH central
midbrain edema central
pontine tegmentum ICH
pontine tegmentum edema
anterior pons ICH
anterior pons edema
cerebellum ICH ipsi
cerebellum edema ipsi
cerebellum ICH contra
cerebellum edema contra
vermis ICH
vermis edema
ICH volume
edema volume
midline shift
intraventricular hemorrhage
propofol
propofol (cumulated)
midazolam
midazolam (cumulated)
dexmed
dexmed (cumulated)
fentanyl
fentanyl (cumulated)
glycemia
BUN
−10.0 −9.6 −9.2 −8.8 −8.4 −8.0 −7.6 −7.2 −6.8 −6.4 −6.0 −5.6 −5.2 −4.8 −4.4 −4.0 −3.6 −3.2 −2.8 −2.4 −2.0 −1.6 −1.2 −0.8 −0.4 0.0 0.4 0.8 1.2 1.6 2.0 2.4 2.8 3.2 3.6 4.0 4.4 4.8 5.2 5.6 6.0 6.4 6.8 7.2 7.6 8.0 8.4 8.8 9.2 9.6 10.0
Parameter weight

1

**Figure S4. Weights of the control model predicting consciousness at time of MRI.**

Include features were: all ROIs with ICH and edema volumes, MLS and IVH plus metabolic and sedation data. Boxplot of all parameter weights obtained over the 500 cross-validation iterations. For scaling visualization purpose outliers are not shown in this figure. Negative weight value corresponds to a predictive value for being unconscious, positive weight corresponds to a predictive value for being conscious. ICH: Intracerebral Hemorrhage; IVH: Intraventricular Hemorrhage; "ipsi" and "contra" stand for ipsilateral and contralateral with respect to the primary side of the hemorrhage; Cx: cortex, BUN: blood Urea Nitrogen.

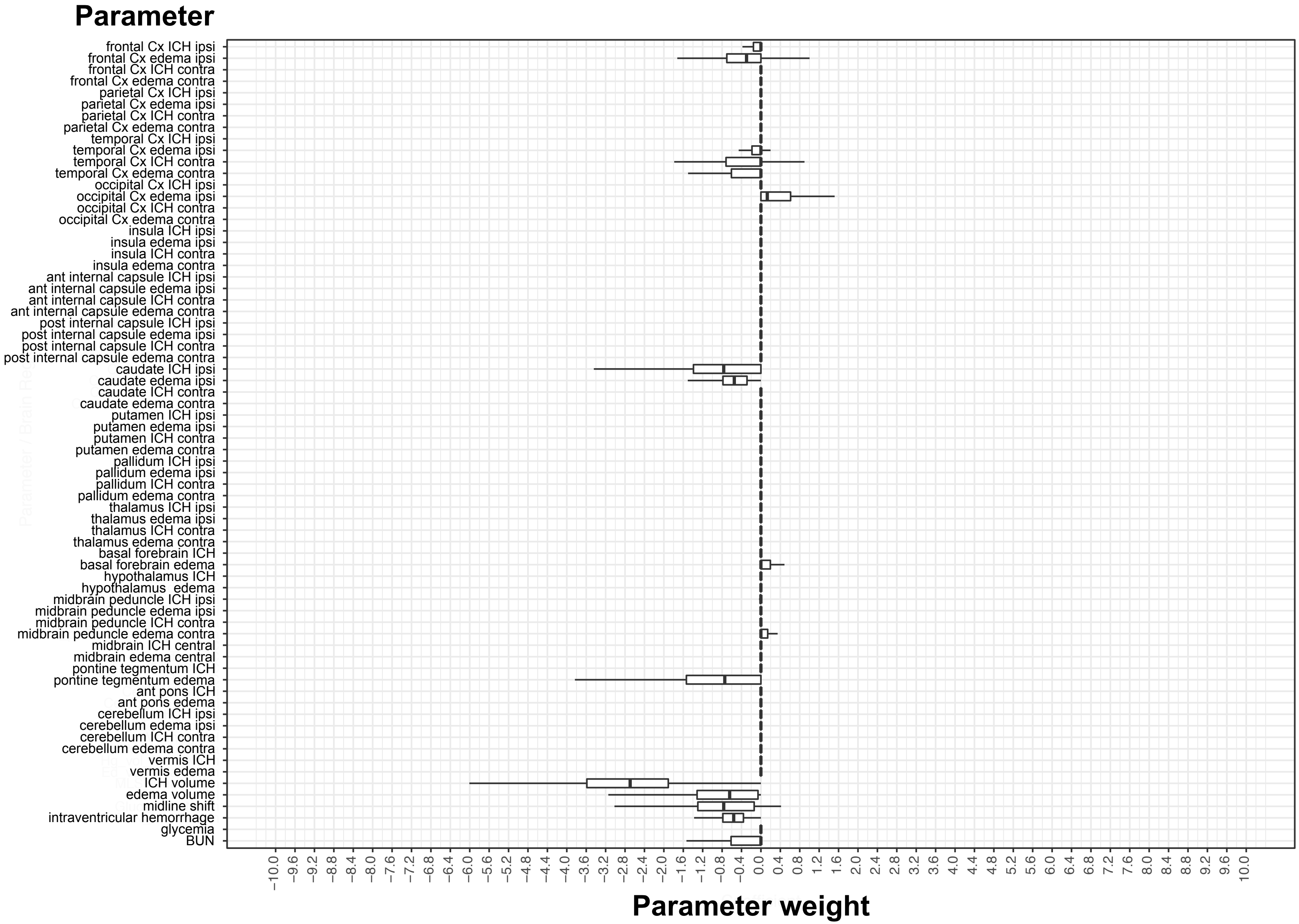
Parameter
frontal Cx ICH ipsi
frontal Cx edema ipsi
frontal Cx ICH contra
frontal Cx edema contra
parietal Cx ICH ipsi
parietal Cx edema ipsi
parietal Cx ICH contra
parietal Cx edema contra
temporal Cx ICH ipsi
temporal Cx edema ipsi
temporal Cx ICH contra
temporal Cx edema contra
occipital Cx ICH ipsi
occipital Cx edema ipsi
occipital Cx ICH contra
occipital Cx edema contra
insula ICH ipsi
insula edema ipsi
insula ICH contra
insula edema contra
ant internal capsule ICH ipsi
ant internal capsule edema ipsi
ant internal capsule ICH contra
ant internal capsule edema contra
post internal capsule ICH ipsi
post internal capsule edema ipsi
post internal capsule ICH contra
post internal capsule edema contra
caudate ICH ipsi
caudate edema ipsi
caudate ICH contra
caudate edema contra
putamen ICH ipsi
putamen edema ipsi
putamen ICH contra
putamen edema contra
pallidum ICH ipsi
pallidum edema ipsi
pallidum ICH contra
pallidum edema contra
thalamus ICH ipsi
thalamus edema ipsi
thalamus ICH contra
thalamus edema contra
basal forebrain ICH
basal forebrain edema
hypothalamus ICH
hypothalamus edema
midbrain peduncle ICH ipsi
midbrain peduncle edema ipsi
midbrain peduncle ICH contra
midbrain peduncle edema contra
midbrain ICH central
midbrain edema central
pontine tegmentum ICH
pontine tegmentum edema
ant pons ICH
ant pons edema
cerebellum ICH ipsi
cerebellum edema ipsi
cerebellum ICH contra
cerebellum edema contra
vermis ICH
vermis edema
ICH volume
edema volume
midline shift
intraventricular hemorrhage
glycemia
BUN
−10.0 −9.6 −9.2 −8.8 −8.4 −8.0 −7.6 −7.2 −6.8 −6.4 −6.0 −5.6 −5.2 −4.8 −4.4 −4.0 −3.6 −3.2 −2.8 −2.4 −2.0 −1.6 −1.2 −0.8 −0.4 0.0 0.4 0.8 1.2 1.6 2.0 2.4 2.8 3.2 3.6 4.0 4.4 4.8 5.2 5.6 6.0 6.4 6.8 7.2 7.6 8.0 8.4 8.8 9.2 9.6 10.0
Parameter weight

1

**Figure S5. Weights of the control model predicting consciousness at time of ICU discharge**

Include features were: all ROIs with ICH and edema volumes, MLS and IVH plus metabolic data. Boxplot of all parameter weights obtained over the 500 cross-validation iterations. For scaling visualization purpose outliers are not shown in this figure. Negative weight value corresponds to a predictive value for being unconscious, positive weight corresponds to a predictive value for being conscious. ICH: Intracerebral Hemorrhage; IVH: Intraventricular Hemorrhage; "ipsi" and "contra" stand for ipsilateral and contralateral with respect to the primary side of the hemorrhage; Cx: cortex, BUN: blood Urea Nitrogen.

**Table S1.** Sedation received at time of MRI.

| | **Conscious (N=105)** | **Unconscious (N=53)** | **p-value** |
|---|---|---|---|
| **Dexmedetomidine** | | | |
| Patients receiving medication, N (%) | 2 (2) | 1 (2) | 1.0 |
| Current dose in mcg/h, mean +/- SD | 31 +/- 17 | 11.2 | 0.67 |
| Patients on medication prior to MRI, N (%) | 3 (3) | 1 (2) | 1.0 |
| Cumulative dose in mcg, mean +/- SD | 93 +/- 80 | 11 | 0.5 |
| **Propofol** | | | |
| Patients receiving medication, N (%) | 6 (6) | 7 (13) | 0.13 |
| Current dose in mg/h, mean +/- SD | 185 +/- 96 | 91 +/- 46 | **0.04** |
| Patients on medication prior to MRI, N (%) | 6 (6) | 10 (19) | **0.02** |
| Cumulative dose in mg, mean +/- SD | 361+/-202 | 131 +/-69 | **0.01** |
| **Midazolam** | | | |
| Patients receiving medication, N (%) | 1 (1) | 1 (2) | 1 |
| Current dose in mg/h, mean +/- SD | 1 | 1 | - |
| Patients on medication prior to MRI, N (%) | 3 (3) | 2 (4) | 1 |
| Cumulative dose in mg, mean +/- SD | 1 +/-1 | 8 +/-1 | 0.14 |
| **Fentanyl** | | | |
| Patients receiving medication, N (%) | 3 (3) | 6 (11) | 0.06 |
| Current dose in mcg/h, mean +/- SD | 33 +/-4 | 33 +/-13 | 0.89 |
| Patients on medication prior to MRI, N (%) | 6 (6) | 8 (15) | 0.08 |
| Cumulative dose in mcg, mean +/- SD | 108 +/-88 | 196 +/-131 | 0.17 |

Cumulative doses over the previous 2 half-lives from MRI behavioral assessment (corresponding to 4h for dexmedetomidine, 2h for propofol, 24h for midazolam and 8h for fentanyl). MRI: Magnetic Resonance Imaging; SD: Standard Deviation.

## Table S2. ICH and Edema prevalence at time of MRI.

| | ICH | | | | Edema | | | |
|---|---|---|---|---|---|---|---|---|
| | Conscious (N=105) | | Unconscious (N=53) | | Conscious (N=105) | | Unconscious (N=53) | |
| | ipsilateral | contralateral | ipsilateral | contralateral | ipsilateral | contralateral | ipsilateral | contralateral |
| Frontal cortex | 24% (N=25) | 1% (N=1) | 47% (N=25) | 6% (N=3) | 35% (N=37) | 1% (N=1) | 62% (N=33) | 8% (N=4) |
| Parietal cortex | 19% (N=20) | 4% (N=4) | 23% (N=12) | 2% (N=1) | 25% (N=26) | 4% (N=4) | 28% (N=15) | 4% (N=2) |
| Temporal cortex | 22% (N=23) | 0% (N=0) | 36% (N=19) | 4% (N=2) | 27% (N=28) | 0% (N=0) | 43% (N=23) | 6% (N=3) |
| Occipital cortex | 8% (N=8) | 1% (N=1) | 2% (N=1) | 2% (N=1) | 10% (N=10) | 1% (N=1) | 4% (N=2) | 2% (N=1) |
| Insula cortex | 9% (N=9) | 0% (N=0) | 15% (N=8) | 0% (N=0) | 9% (N=9) | 0% (N=0) | 17% (N=9) | 0% (N=0) |
| Internal capsule | | | | | | | | |
| Anterior limb | 3% (N=3) | 0% (N=0) | 6% (N=3) | 0% (N=0) | 8% (N=8) | 0% (N=0) | 21% (N=11) | 0% (N=0) |
| Posterior limb | 20% (N=21) | 0% (N=0) | 23% (N=12) | 0% (N=0) | 39% (N=41) | 0% (N=0) | 43% (N=23) | 2% (N=1) |
| **Caudate nuclei** | 5% (N=5) | 0% (N=0) | 9% (N=5) | 0% (N=0) | 9% (N=9) | 1% (N=1) | 23% (N=12) | 0% (N=0) |
| **Putamen** | 20% (N=21) | 0% (N=0) | 26% (N=14) | 2% (N=1) | 33% (N=35) | 0% (N=0) | 40% (N=21) | 2% (N=1) |
| **Pallidum** | 22% (N=23) | 1% (N=1) | 26% (N=14) | 0% (N=0) | 36% (N=38) | 0% (N=0) | 38% (N=20) | 2% (N=1) |
| **Thalamus** | 20% (N=21) | 1% (N=1) | 28% (N=15) | 0% (N=0) | 37% (N=39) | 1% (N=1) | 42% (N=22) | 6% (N=3) |
| **Basal forebrain*** | 0% (N=0) | | 0% (N=0) | | 2% (N=2) | | 13% (N=7) | |
| **Hypothalamus*** | 1% (N=1) | | 2% (N=1) | | 11% (N=12) | | 25% (N=13) | |
| **Midbrain peduncle** | 2% (N=2) | 0% (N=0) | 9% (N=5) | 0% (N=0) | 18% (N=19) | 1% (N=1) | 21% (N=11) | 4% (N=2) |
| **Midbrain central*** | 6% (N=6) | | 8% (N=4) | | 13% (N=14) | | 21% (N=11) | |
| **Pontine tegmentum*** | 5% (N=5) | | 9% (N=5) | | 7% (N=7) | | 15% (N=8) | |
| Anterior pons* | 7% (N=7) | | 9% (N=5) | | 10% (N=10) | | 15% (N=8) | |
| Cerebellum | 13% (N=14) | 3% (N=3) | 2% (N=1) | 2% (N=1) | 11% (N=12) | 6% (N=6) | 4% (N=2) | 4% (N=2) |
| Vermis* | 3% (N=3) | | 0% (N=0) | | 6% (N=6) | | 2% (N=1) | |
| IVH* | 44% (N=46) | | 58% (N=31) | | -- | | -- | |

Bold ROIs correspond to the ROIs of interest (in pink in figure S1).

* These regions are either just central or don't have a side associated with them. ICH: Intracerebral Hemorrhage; IVH: Intraventricular Hemorrhage.

**Table S3. ICH and edema lesion at time of ICU discharge.**

| | **ICH** | | | | **Edema** | | | |
|---|---|---|---|---|---|---|---|---|
| | Conscious (N=125) | | Unconscious (N=33) | | Conscious (N=125) | | Unconscious (N=33) | |
| | ipsilateral | contralateral | ipsilateral | contralateral | ipsilateral | contralateral | ipsilateral | contralateral |
| Frontal cortex | 26% (N=33) | 2% (N=2) | 52% (N=17) | 6% (N=2) | 38% (N=47) | 2% (N=3) | 70% (N=23) | 6% (N=2) |
| Parietal cortex | 20% (N=25) | 3% (N=4) | 21% (N=7) | 3% (N=1) | 24% (N=30) | 3% (N=4) | 33% (N=11) | 6% (N=2) |
| Temporal cortex | 25% (N=31) | 1% (N=1) | 33% (N=11) | 3% (N=1) | 29% (N=36) | 1% (N=1) | 45% (N=15) | 6% (N=2) |
| Occipital cortex | 7% (N=9) | 1% (N=1) | 0% (N=0) | 3% (N=1) | 10% (N=12) | 1% (N=1) | 0% (N=0) | 3% (N=1) |
| Insula cortex | 10% (N=12) | 0% (N=0) | 15% (N=5) | 0% (N=0) | 10% (N=12) | 0% (N=0) | 18% (N=6) | 0% (N=0) |
| Internal capsule | | | | | | | | |
| Anterior limb | 3% (N=4) | 0% (N=0) | 6% (N=2) | 0% (N=0) | 9% (N=11) | 0% (N=0) | 24% (N=8) | 0% (N=0) |
| Posterior limb | 19% (N=24) | 0% (N=0) | 27% (N=9) | 0% (N=0) | 38% (N=47) | 0% (N=0) | 52% (N=17) | 3% (N=1) |
| **Caudate nuclei** | 4% (N=5) | 0% (N=0) | 15% (N=5) | 0% (N=0) | 8% (N=10) | 1% (N=1) | 33% (N=11) | 0% (N=0) |
| **Putamen** | 20% (N=25) | 0% (N=0) | 30% (N=10) | 3% (N=1) | 33% (N=41) | 0% (N=0) | 45% (N=15) | 3% (N=1) |
| **Pallidum** | 22% (N=27) | 1% (N=1) | 30% (N=10) | 0% (N=0) | 35% (N=44) | 0% (N=0) | 42% (N=14) | 3% (N=1) |
| **Thalamus** | 21% (N=26) | 1% (N=1) | 30% (N=10) | 0% (N=0) | 36% (N=45) | 1% (N=1) | 48% (N=16) | 9% (N=3) |
| **Basal forebrain*** | 0% (N=0) | | 0% (N=0) | | 4% (N=5) | | 12% (N=4) | |
| **Hypothalamus*** | 1% (N=1) | | 3% (N=1) | | 14% (N=17) | | 24% (N=8) | |
| **Midbrain peduncle** | 2% (N=2) | 0% (N=0) | 15% (N=5) | 0% (N=0) | 18% (N=23) | 2% (N=3) | 21% (N=7) | 0% (N=0) |
| **Midbrain central*** | 4% (N=5) | | 15% (N=5) | | 14% (N=18) | | 21% (N=7) | |
| **Pontine tegmentum*** | 5% (N=6) | | 2% (N=2) | | 6% (N=8) | | 5% (N=6) | |
| Anterior pons* | 6% (N=7) | | 2% (N=2) | | 10% (N=12) | | 3% (N=4) | |
| Cerebellum | 11% (N=14) | 2% (N=3) | 3% (N=1) | 3% (N=1) | 10% (N=12) | 5% (N=6) | 6% (N=2) | 6% (N=2) |
| Vermis* | 2% (N=3) | | 0% (N=0) | | 5% (N=6) | | 3% (N=1) | |
| IVH* | 41% (N=51) | | 79% (N=26) | | -- | | -- | |

Bold ROIs correspond to the ROIs of interest (in pink in figure S1).
* These regions are either just central or don’t have a side associated with them. ICH: Intracerebral Hemorrhage; IVH: Intraventricular Hemorrhage.

**Table S4.** Univariate comparisons between conscious and unconscious patients at time of MRI and at time of ICU discharge.

| | Clinical assessment at time of MRI | | | Clinical assessment at ICU discharge | | |
|---|---|---|---|---|---|---|
| | Conscious (N=105) | Unconscious (N=53) | OR (95%, CI) | Conscious (N=125) | Unconscious (N=33) | OR (95%, CI) |
| **Admission prediction scores** | | | | | | |
| Primary ICH score | 1 [1, 2] | 2 [1, 3] | 0.44 (0.30, 1.64) | 1 [1, 2] | 3 [2, 3] | **0.21** (0.12, 0.37) |
| FUNC score | 9 [8, 10] | 8 [6, 9] | **1.42** (1.17, 1.73) | 9 [8, 10] | 7 [6, 8] | **1.70** (1.33, 2.16) |
| GCS | 15 [13, 15] | 9 [7, 11] | **1.57** (1.37, 1.80) | 14 [11, 15] | 8 [7, 10] | **1.45** (1.27, 1.66) |
| **MRI metrics** | | | | | | |
| Time ICH to MRI, days | 2 [1, 3] | 2 [1, 3] | 0.91 (0.77, 1.08) | 2 [1, 3] | 2 [1, 3] | 0.91 (0.76, 1.10) |
| Time MRI to ICU discharge, days | 2 [0, 4] | 5 [2, 11] | **0.91** (0.85, 0.96) | 2 [1, 4] | 9 [4, 14] | **0.88** (0.82, 0.94) |
| Lobar | 35 (33) | 21 (40) | 0.76 (0.38, 1.51) | 38 (32) | 18 (47) | 0.81 (0.37, 1.78) |
| Deep | 43 (41) | 19 (36) | 1.24 (0.63, 2.46) | 48 (40) | 14 (37) | 0.62 (0.29, 1.34) |
| Infratentorial | 18 (17.1) | 10 (18.9) | 0.89 (0.38, 2.09) | 19 (15.8) | 9 (23.7) | 0.60 (0.24, 1.51) |
| ICH volume, ml* | 7 [2, 16] | 22 [9, 43] | **0.58** (0.46, 0.73) | 9 [3, 19] | 28 [8, 48] | **0.64** (0.52, 0.80) |
| Edema volume, ml* | 16 [5, 28] | 37 [17, 65] | **0.73** (0.63, 0.85) | 18 [7, 31] | 50 [17, 72] | **0.75** (0.65, 0.86) |
| MLS, mm* | 0 [0, 0] | 2 [0, 6] | **0.73** (0.63, 0.84) | 0 [0, 2] | 0 [0, 8] | **0.79** (0.70, 0.89) |
| IVH | 46 (44) | 31 (58) | 0.55 (0.28, 1.10) | 51 (41) | 26 (79) | **0.19** (0.07, 0.46) |

ICH and edema volumes and MLS were normalized using the patient's individual total brain volume in relation to group mean brain volumes (mean group brain volume = 1451mL). * OR for ICH and edema volumes correspond to 10ml units, OR for MLS correspond to 1mm changes.
Data reported as N (%) or medians [25%-IQR, 75%-IQR] as appropriate.
OR: odd ratio; GCS: Glasgow Coma Scale; ICH: Intra-Cerebral Hemorrhage; MRI: Magnetic Resonance Imaging; ICU: Intensive Care Unit; MLS: Midline Shift; IVH: Intra-Ventricular Hemorrhage.

**Table S5.** Comparison between patients with and without caudate lesions according to the presence of IVH.

| | Conscious at time of MRI | Conscious at time of ICU discharge |
|---|---|---|
| Caudate lesion, IVH* (N=23) | 11 (48%) | 12 (52%) |
| No caudate lesion, IVH (N=56) | 37 (66%) | 41 (73%) |
| No caudate lesion, no IVH (N=79) | 57 (72%) | 72 (91%) |

* 21 out of 23 patients with caudate lesion also had IVH
Data reported as N (%).
